# An Automatic-Segmentation- and Hyper-Parameter-Optimization-Based Artificial Rabbits Algorithm for Leaf Disease Classification

**DOI:** 10.3390/biomimetics8050438

**Published:** 2023-09-19

**Authors:** Ihtiram Raza Khan, M. Siva Sangari, Piyush Kumar Shukla, Aliya Aleryani, Omar Alqahtani, Areej Alasiry, M. Turki-Hadj Alouane

**Affiliations:** 1 Department of Computer Science, Jamia Hamdard, Delhi 110062, India; iraza@jamiahamdard.ac.in; 2 Department of CSE, KPR Institute of Engineering and Technology, Coimbatore 641407, India; sivasangari.m@kpriet.ac.in; 3 Computer Science & Engineering Department, University Institute of Technology, Rajiv Gandhi Proudyogiki Vishwavidyalaya (Technological University of Madhya Pradesh), Bhopal 462033, India; 4 College of Computer Science, King Khalid University, Abha 62529, Saudi Arabia; osalqahtani@kku.edu.sa (O.A.); areej.alasiry@kku.edu.sa (A.A.); malouane@kku.edu.sa (M.T.-H.A.)

**Keywords:** Artificial Rabbits Algorithm, Automatic Segmentation, Hyper Parameter Optimization, leaf disease classification, synthetic images

## Abstract

In recent years, disease attacks have posed continuous threats to agriculture and caused substantial losses in the economy. Thus, early detection and classification could minimize the spread of disease and help to improve yield. Meanwhile, deep learning has emerged as the significant approach to detecting and classifying images. The classification performed using the deep learning approach mainly relies on large datasets to prevent overfitting problems. The Automatic Segmentation and Hyper Parameter Optimization Artificial Rabbits Algorithm (AS-HPOARA) is developed to overcome the above-stated issues. It aims to improve plant leaf disease classification. The Plant Village dataset is used to assess the proposed AS-HPOARA approach. Z-score normalization is performed to normalize the images using the dataset’s mean and standard deviation. Three augmentation techniques are used in this work to balance the training images: rotation, scaling, and translation. Before classification, image augmentation reduces overfitting problems and improves the classification accuracy. Modified UNet employs a more significant number of fully connected layers to better represent deeply buried characteristics; it is considered for segmentation. To convert the images from one domain to another in a paired manner, the classification is performed by HPO-based ARA, where the training data get increased and the statistical bias is eliminated to improve the classification accuracy. The model complexity is minimized by tuning the hyperparameters that reduce the overfitting issue. Accuracy, precision, recall, and F1 score are utilized to analyze AS-HPOARA’s performance. Compared to the existing CGAN-DenseNet121 and RAHC_GAN, the reported results show that the accuracy of AS-HPOARA for ten classes is high at 99.7%.

## 1. Introduction

Agriculture is considered as a significant source of income in African and Asian countries [1]. To perform precise agriculture, plant disease detection and classification play significant roles in producing more yield for farmers and helping to improve their standard of life [2]. Detecting and classifying plant diseases at an early stage can minimize the economic losses of farmers and the nation [3]. In today’s world, tomatoes are the second most liked by people worldwide, but they are susceptible to diseases and pests during their growth, which seriously disturbs their crop and quality [4]. Among these, leaf diseases need to be considered as a significant phase, since leaves help in photosynthesis [5]. Tomatoes are generally affected by various diseases such as leaf curl, spots, mosaic bacterial wilt, early blight, and fruit canker [6] under extreme weather conditions and environmental factors [7]. Identifying and classifying leaf diseases at their early stages improves the yield of tomatoes and minimizes losses [8]. On the other hand, the poor prediction and classification of plant leaf disease leads to overusing pesticides, which affects plant growth and severely impacts crops [9]. Moreover, detecting plant disease with the naked eye creates complexity for farmers and consumes more time, resulting in degradation in the quality of plants [10].

Approaches based on Artificial Intelligence (AI) can minimize errors while classifying the images of diseased plant leaves [11]. In recent years, approaches based on deep learning techniques have helped researchers who work in the agricultural domain to classify images of disease-affected leaves [12]. Practical classification can be achieved using effective image augmentation, where the images are augmented and new training samples are created from the existing ones [13]. Augmentation methods generate converted versions of the images from the dataset to improve diversity [14]. However, traditional augmentation approaches face challenges and provide a higher misclassification ratio [15]. Using deep learning approaches for classification offers better results than machine learning approaches [16,17]. In current research, standard image augmentation techniques such as shift, zoom, and rotation are used to generate new images from original datasets and increase the number of images. Still, they are unable to decrease misclassifications [18,19].

Additionally, the methods currently in use based on tomato leaf disease are ineffective, resulting in misclassification with a lower accuracy [20]. The aforementioned problem is the impetus for this study’s effort to create a successful augmentation strategy for enhancing classification performance. According to this research, AS-HPOARA improves classification accuracy by enhancing the image with a pixel-by-pixel residual method. Additionally, the proposed method tends to learn and anticipate the ideal size and form of each pixel in the leaf image.

The key contributions are as follows:

The data were initially gathered from the Plant Leaf Disease dataset, where rotation, scaling, and translation were employed to stabilize the training images and z-score normalization was used to normalize the images.

Deep learning data segmentation based on Modified UNet was created for producing effective augmented images at different resolutions. Modified UNet employs a more significant number of fully connected layers to generate a better representation of deeply buried characteristics.

In addition, the plant leaf images were classified using the augmented images created from the source images. The classification was carried out using HPO-based ARA, where the training data were enhanced and the statistical bias was eliminated to raise the classification accuracy in order to convert the images from one domain to another in a paired manner. 

Additionally, the model complexity was reduced by adjusting the hyperparameters that minimize overfitting. Lastly, plant leaf diseases were categorized using the HPOARA system.

This research is arranged as follows: Section 2 provides related works about data augmentation and classification techniques developed for plant disease detection. A detailed explanation of AS-HPOARA is provided in Section 3, whereas the outcomes of the AS-HPOARA are specified in Section 4. Further, conclusions are made in Section 5.

## 2. Related Work

Wu [21] used GAN-based data augmentation to improve the categorization of tomato leaf disease. The Deep Convolutional GAN (DCGAN) was created to create enhanced images, and GoogleNet was utilized to forecast diseases. The DCGAN was optimized using the learning rate, batch size, and momentum to produce more realistic and diverse data. The use of noise-to-image GANs, meanwhile, that portray the image of healthy leaves as ill leaves, led to an imbalance in effectiveness. 

Abbas [22] described a deep learning method that produced synthetic images of plant leaves using Conditional GAN (C-GAN). An additional identification of tomato diseases was performed using the generated synthetic images. Additionally, the DenseNet121 was trained to classify tomato leaf diseases using fake and real images. However, based on only appearance, the C-GAN could not identify different disease stages.

Sasikala Vallabhajosyula et al. [23] presented a Deep Ensemble Neural Network (DENN) based on transfer learning to detect plant leaf disease. While tuning the hyper-parameters, the authors of this research hoped to enhance the classification utilizing DENN and transfer learning. With the aid of transfer learning, these models were confident in extracting discriminating features. The suggested algorithms accurately classified plant leaf diseases by extracting the distinguishing characteristics from leaves. The plant pathologists found it difficult and time-consuming to identify plant diseases manually, and this method was unreliable.

A unique, 14-layered deep convolutional neural network (14-DCNN) was presented by J. Arun Pandian et al. [24] to identify plant leaf diseases from leaves. Several open datasets were combined to form a new dataset. The dataset’s class sizes were balanced using data augmentation techniques. One thousand training epochs of the suggested DCNN model were conducted in an environment with multiple graphics processing units (GPUs). The most appropriate hyperparameter values were chosen randomly using the coarse-to-fine searching strategy to enhance the proposed DCNN model’s training efficacy. Additional data were needed for the DCNN’s training procedure to be effective.

The fine-grained-GAN was introduced by Zhou [25] to perform local spot area data augmentation. To improve the identification of grape leaf spots, data augmentation was used. It utilized hierarchical mask generation to increase the ability of spot feature representation. An upgraded quick R-CNN and fine-grained-GAN were integrated with a fixed-size bounding box to reduce computations and prevent the classifier’s scale variability. However, the technique offered was only appropriate for finding visible leaf spots.

A collaborative framework of diminished features and effective feature selection for cucumber leaf disease identification was offered by Jaweria Kianat et al. [26]. This study proposed a hybrid structure built on feature fusion and selection algorithms that used three fundamental phases to categorize cucumber disease. At last, a collection of classifiers was used to categorize the most discriminant traits. During the serial-based fusion stage, better features over a threshold were chosen. However, it was time-consuming, challenging, prone to mistakes, and deceptive.

To expand the data on tomato leaves and identify diseases, Deng [27] built the RAHC_GAN. For adjusting the size of the actual disease region and enhancing the intra-class data, hidden parameters were included in the input side of the generator. Additionally, residual attention block was included to help the disease region concentrate better. Next, a multi-scale discriminator was employed to enhance the texture of the newly produced images. However, GAN introduced variability while identifying the images, which reduced the overall effectiveness.

Multi-objective image segmentation was used to show tea leaf disease identification by Somnath Mukhopadhyay et al. [28]. The Non-dominated Sorting Genetic Algorithm (NSGA-II) for image clustering was suggested for finding the disease area in tea leaves. Next, the tea leaves’ corresponding feature reduction and disease identification were accomplished using PCA and a multi-class SVM. To fully assist farmers, the suggested system recognized five different diseases from the input and offered relevant actions to be performed. A higher accuracy was achieved by this method; however, it was very time-consuming.

Plant disease classification using ARO with an improved deep learning model was demonstrated by K. Jayaprakash and Dr. S. P. Balamurugan [29]. The developed AROIDL-PDC technique aims to identify and classify various plant diseases. The AROIDL-PDC technique employs a median filtering (MF) strategy during preprocessing. An upgraded version of the MobileNeXt algorithm was also used for feature extraction. The procedure of hyperparameter tuning was then carried out using the ARO approach. Finally, the logistic regression (LR) classifier categorized plant diseases. Several simulations were run to show how the AROIDL-PDC technique performed better.

Deep neural networks through transfer learning were used to forecast rice leaf diseases [30]. In this study, an InceptionResNetV2 model that had already been trained contained the information as weights, which were then transferred to the research investigation for the feature extraction process utilizing the transfer learning approach. Deep learning was enhanced to increase the accuracy in classifying the many diseases affecting rice leaves. The accuracy was improved by running 15 epochs of the simple CNN model with various hyperparameters to 84.75%.

## 3. Proposed AS-HPOARA Method

**Figure 1:**
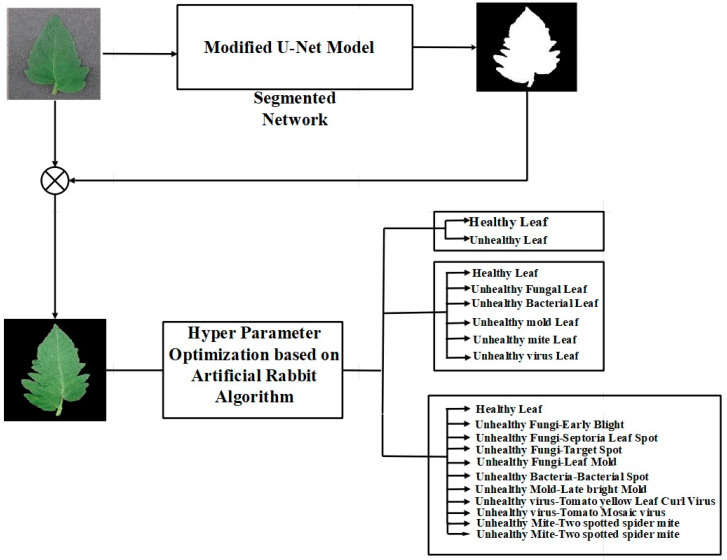
Block diagram for the overall AS-HPOARA method.

This study aimed to enhance the classification of plant diseases using AS-HPOARA-based data augmentation. The AS-HPOARA method was separated into two parts: the generation of synthetic images using AS-HPOARA and the discriminator-based classification of images of plant diseases. Figure 1 presents a block diagram for the entire AS-HPOARA technique. The generator module received the input images and added a label and an appropriate quantity of noise to produce the pixel variations. The Gaussian Noise data augmentation tool added Gaussian noise to the training images. The sigma value was directly related to the size of the Gaussian Noise effect. 

Additionally, this AS-HPOARA offered enhanced pictures, which improved accuracy. Three different types of classifications were performed in this work: (a) the binary classification of healthy and diseased leaves, (b) the five-class classification of healthy and four diseased leaves, and finally, (c) ten-class classification with healthy and nine different disease classes. All the images were divided into ten different classes, where one class was healthy and the other nine classes were unhealthy. Those unhealthy classes were categorized into five subgroups (namely bacterial, viral, fungal, mold, and mite disease). Some sample tomato leaf images for healthy and different unhealthy classes and leaf masks from the Plant Village dataset are shown in Figure 1.

The Plant Village dataset, which contains leaf images and accompanying segmented leaf masks, was used for this investigation. The optimum segmentation network for separating the leaves from the background was investigated using Modified UNet segmentation algorithms. The Score-Cam visualization, which has proven to be quite trustworthy in diverse applications [31], was employed to validate further the segmented leaf leveraging in the categorization.

### 3.1. Dataset Acquisition

**Figure 2:**
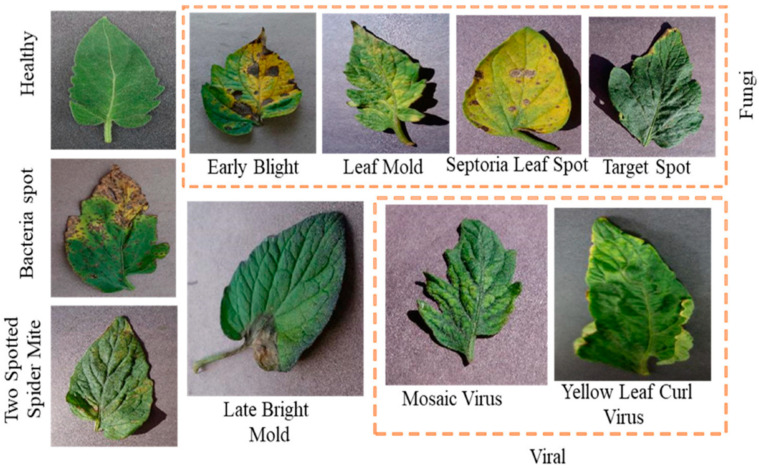
Model images of healthy and unhealthy leaves.

The data utilized in this study to evaluate the AS-HPOARA approach were obtained from the publicly accessible tomato PlantVillage dataset [32]. There are ten different classes in this dataset of 18,161 leaves from PlantVillage. Nine of those ten classes are for diseases, while the final lesson is for health. The ten classes are Tomato Healthy (TH), Tomato Mosaic Virus (TMV), Tomato Early Blight (TEB), Tomato Late Blight (TLB), Tomato Bacterial Spot (TBS), Tomato Leaf Mold (TLM), Tomato Septoria Leaf Spot (TSLS), Tomato Target Spot (TTS), Tomato Yellow Leaf Curl Virus (TYLCV), and Tomato Two-Spotted Spider Mite (TTSSM). Plant disease identification may be performed using the PlantVillage dataset. Illness control procedures can waste time and money and result in additional plant losses if the illness and its causative agent are not correctly identified [33]. Therefore, an accurate illness diagnosis is essential. Plant pathologists frequently have to rely on symptoms to pinpoint a disease issue. It can be used to determine a plant’s species and any diseases it could be carrying. Six augmentation methods, including scaling, rotation, noise injection, gamma correction, picture flipping, and PCA color augmentation, were used to create this dataset [34]. These methods enhanced the dataset to produce a diversified dataset with various background circumstances. Figure 2 displays sample images for the healthy and unhealthy classes and leaf masks. Additionally, Table 1 provides a thorough breakdown of the dataset’s picture count, which is helpful for classification tasks.

The images obtained from the tomato plant village dataset were resampled into the size of 224×224, aiming to improve the classifier’s computational ability. The imbalance problem was avoided using 300 randomly chosen images from each dataset class. Three hundred images were therefore taken into consideration for evaluation over a total of 10 classifications [35]. About 12,000 images were acquired and combined with the real images in the dataset during the final augmentation stage utilizing AS-HPOARA. Thus, a total of 15,000 images were used for the classification, and the dataset was separated into training, validation, and testing phases in the ratio of 70:15:15.

### 3.2. Preprocessing

Z-score normalization was performed to normalize the images using the mean and standard deviation of the dataset’s images [36]. To accommodate the Modified UNet segmentation model, the images were enlarged to 256 × 256 and 224 × 224, respectively.

### 3.3. Augmentation

Training with an unbalanced dataset affects the bias, because the dataset is unbalanced and cannot have an identical number of images for each category. Three augmentation techniques were used in the present investigation to equalize the training images: 

Rotation: The position of an object in the frame is altered by randomly rotating a source image by a certain number of degrees, either clockwise or anticlockwise. As part of the image augmentation procedure, the photos were rotated between 5 and 15 degrees in clockwise and anticlockwise orientations. In this work, 2.5% to 10% image magnifications were used. 

Scaling: Scaling means increasing or decreasing an image’s frame size. In this augmentation technique, the small size of an image within a dimension range is selected at random. This augmentation technique has applications in object detection tasks, for example.

Translation: In order to enhance back translation, text material must first be translated into another language and then back into the original language. This method enables the creation of textual data that differ from the original text’s original context and meaning. The images’ translations in the horizontal and vertical directions ranged from 5% to 20%.

### 3.4. Segmentation 

There are numerous segmentation models using U-nets in research [37]. In the current research, variations of the Modified U-Net [38] were examined to select the one that performed the best.

**Figure 3:**
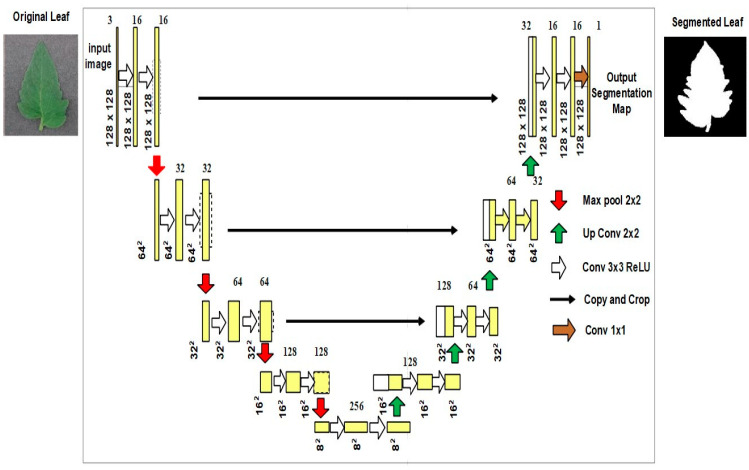
Architecture of Modified U-Net model.

The structure of the Modified U-Net is revealed in Figure 3.

The Modified U-Net was used, a variant of the U-Net model with minor differences in the decoding section. A down sampling max pooling layer with a stride equal to 2 followed each pair of subsequent 3 × 3 convolutional layers in an encoding block. All the convolutional layers were expanded to use batch normalization and ReLU activation. At the last layer, a pixel-by-pixel SoftMax was used to convert every pixel into a class of binary backdrop. This layer then employed 1 × 1 convolution for translating the output from the final decoding block to feature maps.

#### 3.4.1. Hyperparameter Optimization Based Classification

The classification performance was improved by adjusting the Artificial Rabbits Algorithm (ARA)’s (hyperparameter optimization) ideal hyperparameters. Regulating the learning behavior of the constructed models involved optimizing the hyperparameters. The established model parameters produced satisfactory results if the hyperparameters were appropriately tuned, since they did not minimize the loss function. To achieve the best classification results, hyperparameter optimization was performed. This work used hyperparameter-optimization-based ARA for classification [39]. The below steps reveal the search processes of the ARA approach.

#### 3.4.2. Detour Foraging (Exploration)

When foraging, rabbits prefer to wander to far regions where other people are, neglecting what is nearby, much like an old Chinese proverb that states [40]: “A rabbit does not eat grass near its own nest.” This is called detour foraging, and its numerical equation is displayed in Equations (1)–(5),

(1) $$X_{i}\left(t+1\right)=X_{j}\left(t\right)+A\times (X_{i}\left(t\right)-X_{j}\left(t\right)+round\left(0.5\times R_{1}\right))\times n_{1}$$

(2) A=L×c

(3) $$L=\left(e-e^{(\frac{t-1}{T}{)}^{2}}\right)\times sinsin\;(2\pi R_{2})\;$$

(4) g=ransperm (D)

(5) $$n_{1}\sim \;N(0,1)$$

where,

$X_{i}\left(t+1\right)\to candidate\;position\;of\;the\;i-th\;rabbit\;in\;iteration$
$X_{j}\left(t\right)\to Position\;of\;i-th\;rabbit$
N→Denotes the population size
t →Maximum number of iterations
D →Dimension size
round → rounding to the nearest integer,
ransperm → returns a random permutation of integers,
$R_{1},\;R_{2}$ → random numbers
L →Running length
$n_{1}\to$ standard normal distribution

#### 3.4.3. Transition from Exploration to Exploitation

It has already made the move from exploration, and it will continue to do so over time. The following formula describes the power factor E: In ARA, rabbits frequently adopt random concealment in the later stages of the search, while they are more likely to use continuous detour foraging in the early stages of the iteration. Equation (6) illustrates the idea of rabbit energy E to achieve a balanced ratio between exploitation and exploration.

(6) $$E\left(t\right)=4\;\left(1-\frac{t}{T}\;\right)In\;\frac{1}{R_{4}}\;$$

#### 3.4.4. Random Hiding (Exploitation)

Predators commonly pursue and attack rabbits. To survive, they would dig a variety of shelter-filled burrows all around the nest. In ARA [41], a rabbit constantly builds D burrows throughout the search space’s dimensions and then chooses one at random to hide in to reduce the likelihood of being caught. The mathematical model of this behavior is illustrated in Equations (7)–(11):

(7) $$X_{i}\left(t+1\right)=X_{i}\left(t\right)+A\times (R_{5}\times b_{i,r}\left(t\right)-X_{i}\left(t\right))$$

(8) $$b_{i,r}\left(t\right)=X_{i}\left(t\right)+H\times g_{r}\;\left(k\right)\times X_{i}(t)$$

(9) $$g_{r}\;\left(k\right)=\{1,\;if\;k==[R_{6}\times D]\;0,\;\;\;otherwise\;$$

(10) $$H=\frac{T-t+1}{T}\times n_{2}\;$$

(11) $$n_{2}\sim \;N\left(0,1\right)\;\;\;\;\;\;\;\;\;\;\;$$

$\;b_{i,r}\left(t\right)$ depicts the i-th rabbit’s burrow randomly. H refers to the hiding parameter. In the present version, burrows are utilised for hiding t, $R_{5}$ and $R_{6}$ are two random numbers between 0 and 1, and $n_{2}$ follows a standard normal distribution [42].

## 4. Results and Discussion

The proposed technique was modeled by Python 3.7, and the system was run with parameters such as 8GB RAM and an i5 processor. In this section, the AS-HPOARA method’s results are explained. It is suggested to improve the classification of tomato leaf diseases using AS-HPOARA-based data augmentation and classification. 

### 4.1. Leaf Segmentation Analysis 

The test set’s class distribution resembled that of the training set. In order to attain the most significant performance measures and choose the optimal tomato leaf segmentation model, three distinct loss functions—Negative Log-Likelihood (NLL) loss, Binary Cross-Entropy (BCE) loss, and Mean-Squared Error (MSE) loss—were utilized in the present investigation. Additionally, as described in several recent publications, an initial termination standard of five epochs with no enhancement in validation loss was adopted. Table 2 compares the presentations of the segmentation model while utilizing the NLL, BCE, and MSE loss functions as three distinct types of segmentation loss functions. It should be highlighted that the Modified UNet with NLL performed well in both quantitative and qualitative terms when segmenting the leaf region over the entire set of images, in terms of test loss (0.0076), test accuracy (98.66), and dice (98.73) for the segmentation of tomato leaves, respectively.

The Modified U-net model was applied to the specified dataset to find the top-performing leaf segmentation model. Five-fold cross-validation was used, where 70% of the leaf images and their corresponding ground truth masks were randomly selected and used for training, the remaining 15% for testing, and another 15% for validation. The test set’s class distribution resembled that of the training set. To avoid the overfitting issue, 90% of the training dataset comprising 70% of the dataset was used for the training, while 10% was used for the validation. The entire training and inference pipelines were described using k-fold cross-validation (2, 3, 4, and 5). Table 3 shows the k-fold validation for the ten classes with NLL loss functions, which provided better results when compared to the BCE and MSE loss functions.

### 4.2. Leaf Disease Classification Analysis

In order to classify the segmented portions of tomato leaf disease, the research looked at a deep learning framework based on a CNN designated as Modified UNet. In this investigation, three distinct categorization trials were run. Table 4 gives an outline of the classification and segmentation trials’ variables. 

An overview of the dataset training and outcomes based on effectiveness and a comparative evaluation are provided in the sections that follow. Additionally, the testing time per image, that is, the amount of time it took for each network to categorize or segment an input image, was additionally contrasted between the segmentation and classification networks. The Modified UNet surpassed the other learned models between the networks trained on leaf pictures with/without segmented 2, 6, and 10-class issues. Additionally, it can be seen that, as the Modified UNet model was scaled, the network’s scaled depth, width, and resolution caused an increase in the testing time. The performance improved whenever the network increased according to authors’ testing of the various Modified UNet versions. The efficiency using the enlarged version of Modified UNet did not improve much as the classification strategy became more complex.

**Figure 4:**
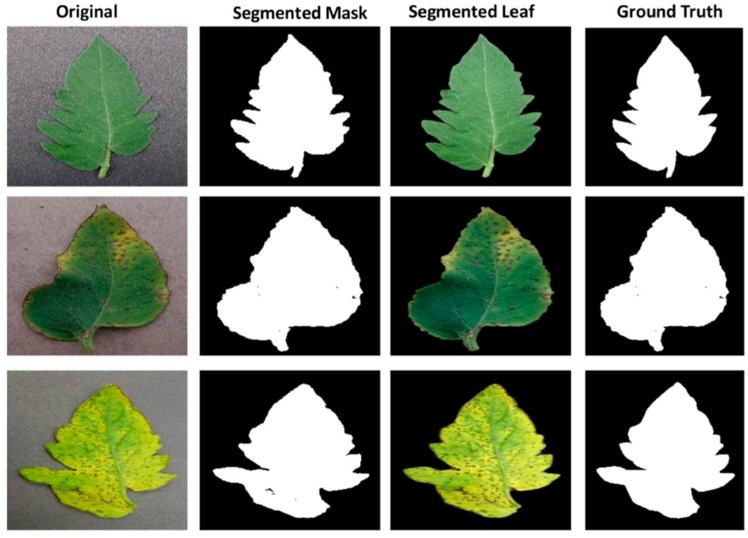
Samples of tomato leaf images.

Figure 4 clearly shows that adding more parameters resulted in better network performances for the 2, 6, and 10-class tasks. For the Plant Village dataset, the Modified U-net using the NLL loss function produced the segmented leaf pictures shown in Figure 4, along with some sample images, related ground truth masks, and test tomato leaf images.

### 4.3. Performance Evaluation of AS-HPOARA

The performance of the proposed AS-HPOARA was assessed by considering the following cases:

Case 1: The binary classes, such as healthy and unhealthy leaves, were considered for analysis.

Case 2: The six different classes were healthy tomato, tomato septoria leaf spot, tomato bacterial spot, tomato late blight, tomato target spot, and tomato yellow leaf curl virus.

Case 3: In this case, all the ten classes obtained from the dataset were considered. 

The performance of AS-HPOARA without data augmentation for all three cases is given in Table 5. While considering the loss function, NLL outperformed the other two loss functions (BCE and MSE), which is clearly mentioned in Table 2. Therefore, Table 6 provides the performance analyses of the different classes for the NLL loss function.

**Figure 5:**
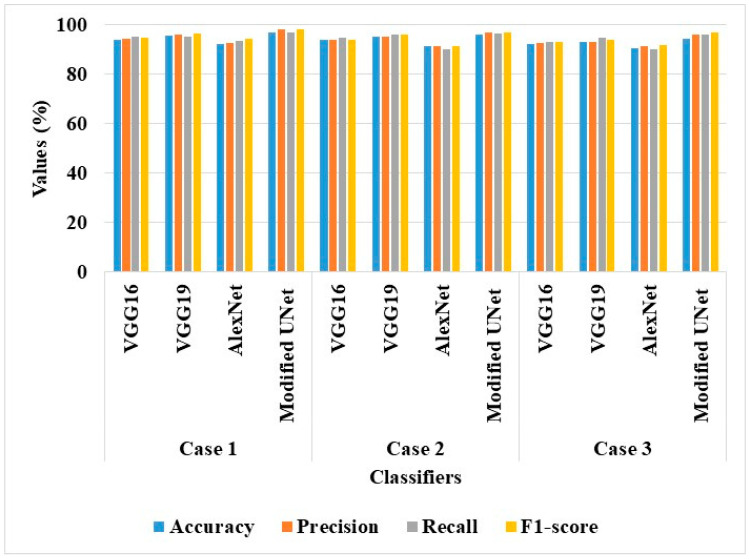
Graphical results of classifiers without AS-HPOARA.

The effectiveness of AS-HPOARA was assessed for the aforementioned scenarios using a variety of classifiers, including VGG16, VGG19, and AlexNet. In this section, both classifiers with and without AS-HPOARA were evaluated for their performances. Next, Figure 5 provides a graphic representation of all the performances.

According to the analysis, in all three scenarios, DenseNet121 without AS-HPOARA offered a greater level of classification accuracy than VGG16, VGG19, and AlexNet. For instance, the Modified UNet achieved an accuracy of 97.11% in case 1, compared to VGG16’s 94.08%, VGG19’s 95.79%, and AlexNet’s 92.36%. Because of its large number of completely linked layers, which aided in achieving a richer representation of deeply buried characteristics, the DenseNet121 performed a better classification. 

Table 6 shows the suggested AS-HPOARA-based data augmentation and performance analysis of the various classifiers (VGG16, VGG19, AlexNet, and Modified UNet). 

**Figure 6:**
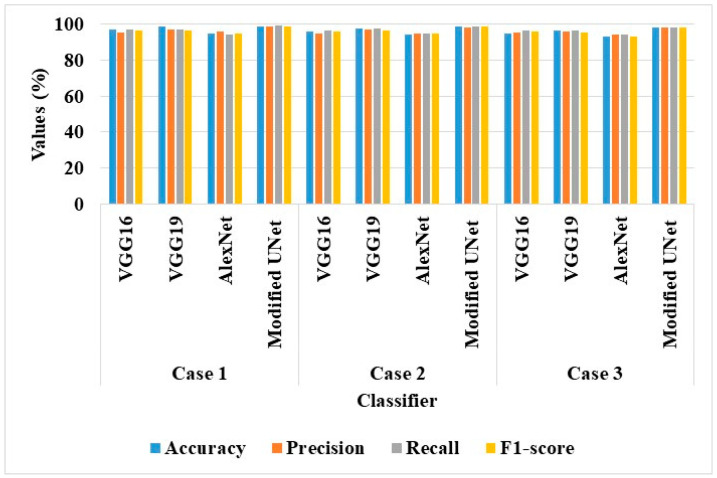
Graphical results of classifiers with AS-HPOARA.

The investigation led to the conclusion that the AS-HPOARA and Modified UNet combo performed better than the other classifiers. For instance, AS-HPOARA achieved an accuracy of 99.08% for case 1, compared to VGG16’s 96.93%, VGG19’s 98.82%, and AlexNet’s 95.08%. Additionally, AS-HPOARA’s accuracy was greater than the accuracy in each of the three scenarios. The discriminator received as an input the augmented images of the various resolutions produced by the AS-HPOARA generator. Consequently, the AS-HPOARA’s progressive training was employed to improve the classification of tomato leaf diseases. Figure 6 displays the graphical outcomes of the classifiers using AS-HPOARA.

### 4.4. Visualization Using Score-Cam

**Figure 7:**
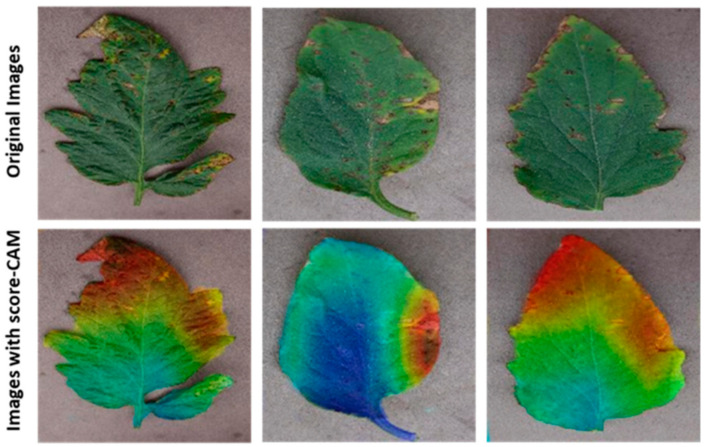
Score-CAM visualization of classified images.

**Figure 8:**
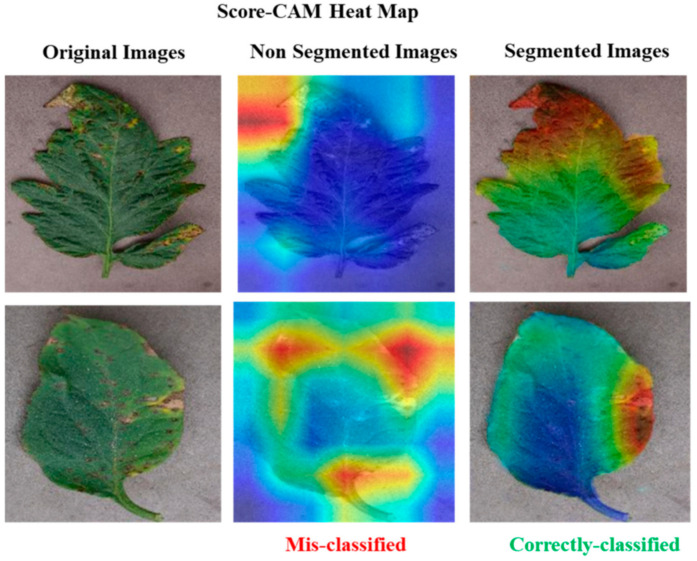
Score-CAM visualization of classified portions.

This study used visualization techniques to examine the trained networks’ dependability. Five separate categories of score-CAM were incorrectly categorized as healthy or harmful classes. Heat maps for the segmented tomato leaf pictures were employed for the ten-class challenge to show the affected portions clearly. Additionally, the networks picked up knowledge from the segmented leaf images, increasing the reliability of the network’s judgment. This served to refute the charge that CNN lacks credibility and draws its decisions from irrelevant regions. Additionally, segmentation aided the categorization, as the network picked up knowledge from the area of interest. This trustworthy education helped in inaccurate categorization. The segmented leaf images leveraging in the classification were supplementarily proved by the Score-Cam visualization procedure, which has been found to be dependable in various applications. Figure 7 displays the segmented leaves’ heat maps and the original tomato leaf samples taken from Python. While Figure 8 shows the Score-CAM visualization of classified portions.

Score-CAM [40] is a recently proposed visualization technique that was utilized in this study because of its promising results among the many visualization techniques that are now accessible, including Smooth Grad, Grad-CAM, Grad-CAM++, and Score-CAM. Each heat map’s weight was determined by its forward passing score on the target class, and the final result was produced by linearly combining the weights and activation maps. By deriving the weight of each activation map from its forward passing score on the target class, Score-CAM eliminated the dependence on gradients. If it can be confirmed that the network always bases its decisions on the leaf area, it can assist users in understanding how the network makes decisions and increase end-user trust.

### 4.5. Comparative Analysis

**Figure 9:**
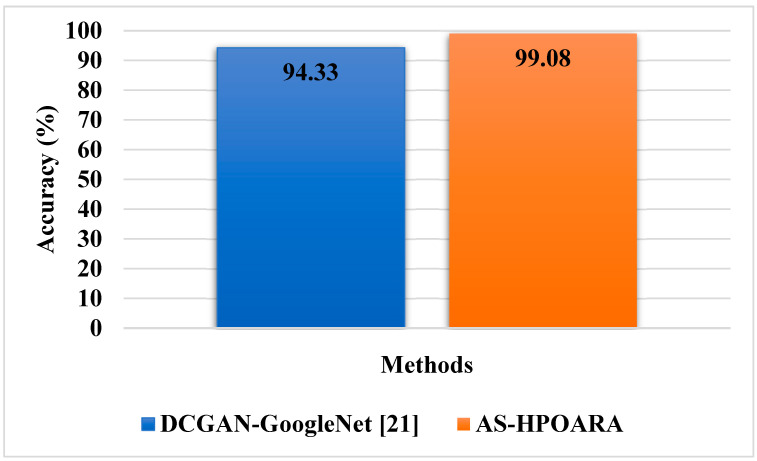
Graphical comparisons of accuracy for AS-HPOARA [21].

In this sub-section, existing methodologies such as Deep Convolutional GAN (DCGAN)-GoogleNet [21] and Conditional GAN (CGAN)-DenseNet121 [22] were utilized to assess the effectiveness of the proposed AS-HPOARA. The DCGAN-GoogleNet was assessed using case 1, and the CGAN-DenseNet121 was evaluated using case 3. The comparison of AS-HPOARA based on case 1 and case 3 is represented in Table 7 and Table 8, respectively. Moreover, the graphical representation for classification accuracy compared with the existing DCGAN-GoogleNet and CGAN-DenseNet121 is presented in Figure 9.

**Figure 10:**
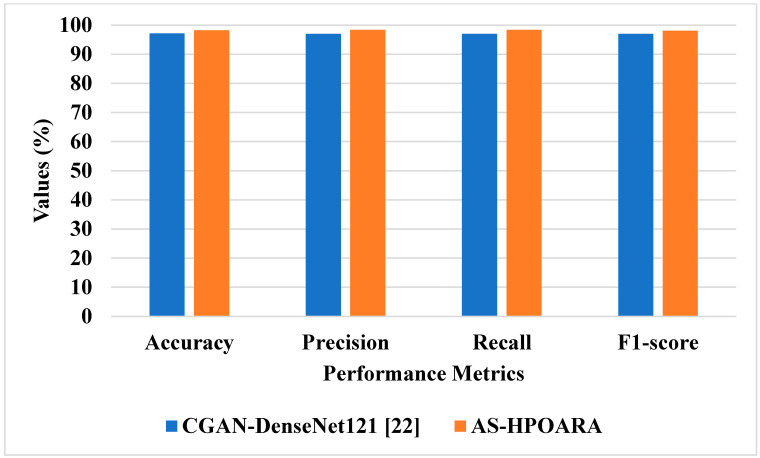
Graphical comparisons of AS-HPOARA and existing CGAN-DenseNet121 [22].

The graphic comparison of the AS-HPOARA and pre-existing CGAN-DenseNet121 is shown in Figure 10. The overall results indicated that, compared to the existing methodologies, the proposed strategy outperformed them regarding overall metrics. For instance, the suggested AS-HPOARA’s classification accuracy for case 1 was 99.08%, compared to the accuracy of DCGAN-Google Net [21] being 94.33%. Like instance 2, the suggested approach had a classification accuracy of 98.7% compared to the existing CGAN-DenseNet121 [22], with an accuracy of 97.11%. Due to its capacity to execute pixelwise distribution to calculate the optimistic shape for every pixel of the leaf picture, the suggested approach produced superior results and improved disease classification. Table 9 shows a comparative analysis of the existing RAHC_GAN [27] regarding accuracy. 

**Figure 11:**
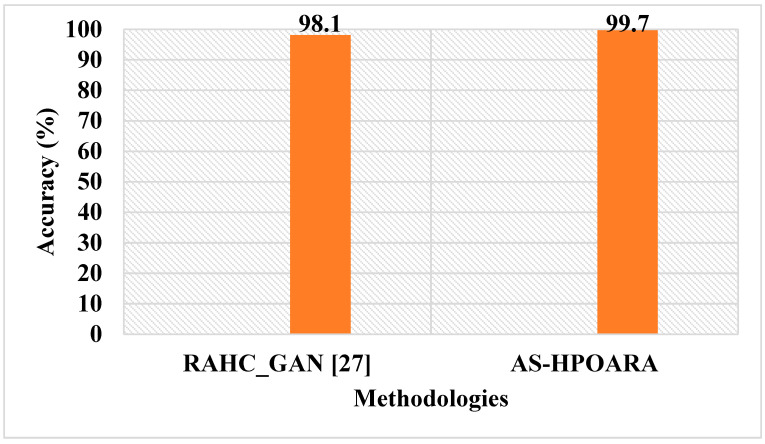
Graphical comparisons of AS-HPOARA and existing RAHC_GAN [27].

Table 9 clearly shows that the proposed AS-HPOARA achieved an accuracy of 99.7%, which was better than the existing RAHC_GAN [27], which had a 98.1% accuracy. A graphical comparison of the AS-HPOARA and the existing RAHC_GAN [27] is shown in Figure 11.

### 4.6. Discussion

This research analyzed different existing techniques; they were DCGAN-GoogleNet [21], CGAN-DenseNet121 [22], and RAHC_GAN [27]. The existing DCGAN-GoogleNet [21] was analyzed using five classes: healthy tomato, tomato late blight, tomato septoria leaf spot, tomato target spot, and tomato yellow leaf curl virus, while in the existing CGAN-DenseNet121 [22], all the ten classes obtained from the dataset were considered. The Automatic Segmentation and Hyper Parameter Optimization based Artificial Rabbits Algorithm (AS-HPOARA) was created to improve the classification of plant leaf diseases. The proposed AS-HPOARA method was evaluated using the PlantVillage dataset. The images were normalized using the dataset’s mean and standard deviation via z-score normalization. The training images were balanced in this work using three augmentation techniques: rotation, scaling, and translation. Image augmentation before classification lowered the overfitting issues and increased the classification precision. The HPO-based ARA performed the classification to convert the images from one domain to another in a pairing manner. The performance of AS-HPOARA was evaluated using accuracy, precision, recall, and F1 score. From the result analysis, the accuracy of AS-HPOARA for ten classes was high at 99.08% compared to the existing DCGAN-GoogleNet [21], which was 94.33%. Subsequently, traditional augmentation only modifies the location and direction of an image, so little data are learned and an enhancement in accuracy is restricted, whereas, taking the existing CGAN-DenseNet121 [22], it obtained an accuracy of 94.33%, a precision of 97%, a recall of 97%, and an F1 score of 97%. In this case, the proposed AS-HPOARA accomplished a better accuracy (98.7%), precision (98.52%), recall (98.58%), and F1 score (98.27%). The existing RAHC_GAN [27] obtained an accuracy of 98.1%, which was much less when compared to the proposed AS-HPOARA, which achieved a 99.7% accuracy.

## 5. Conclusions

The applications of deep learning techniques play a vital role in the computerized classification of leaf diseases. However, overfitting and inadequate data training complicate the current methods for detecting and classifying sick leaves. The PlantVillage dataset was used to assess the proposed AS-HPOARA approach. Z-score normalization was performed using the dataset’s mean and standard deviation to normalize the images. Three augmentations were employed in this study to stabilize the training images: rotation, scaling, and translation. Since Modified UNet uses more fully connected layers to better represent deeply buried features, it was considered for segmentation. In order to translate the images from one domain to another in a paired fashion and assess the uncertainty with the resulting images, the classification was completed using HPO-based ARA. According to the experimental findings, the suggested AS-HPOARA offered superior classification outcomes to the conventional DCGAN-GoogleNet and CGAN-DenseNet121. With a classification accuracy of 99.08% for ten classes, the proposed AS-HPOARA strategy exceeded earlier methods. The proposed AS-HPOARA accomplished an accuracy of 99.7%, while the existing RAHC_GAN achieved an accuracy of 98.1%, which is very low. Additionally, investigating CNN through non-linear feature extraction layers may be beneficial for finding possible results. In the future, this research will be further extended by analyzing various meta-heuristic algorithms to improve the accuracy of leaf disease classification.

## Figures and Tables

**Table 1:** Overall images of the Plant Village dataset.

Class	Healthy	Fungi	Bacteria	Mold	Virus	Mite
Sub Class	Healthy (1591)	Early Blight (1000) Septoria Leaf Spot (1771) Target spot (1404) Leaf mold (952)	Bacterial spot (2127)	Late bright mold (1910)	Tomato Yellow Leaf Curl Virus (5357) Tomato Mosaic Virus (373)	Two-spotted spider mite (1676)
Tomato (18,161)						

**Table 2:** Loss function analysis of modified UNet.

Loss Function	Training Function		Test Function		Validation Function		Dice	Inference Time (s)
	Loss	Accuracy	Loss	Accuracy	Loss	Accuracy		
NLL	0.0063	98.89	0.0076	98.66	0.0079	98.67	98.73	12.12
BCE	0.019	98.25	0.021	98.13	0.023	98.15	97.1	12.04
MSE	0.086	98.27	0.089	98.19	0.091	98.22	98.43	11.76

**Table 3:** K-fold validation for 10-classes with NLL loss function.

K-Fold	Classifier with AS-HPOARA	Accuracy (%)	Precision (%)	Recall (%)	F1-Score (%)
K = 2	VGG16	95.34	95.67	96.55	96.10
	VGG19	98.45	98.56	98.06	98.30
	AlexNet	97.78	97.06	97.45	97.25
	Modified UNet	99.45	98.05	98.45	98.24
K = 3	VGG16	99.67	98.55	98.67	98.60
	VGG19	98.28	97.67	98.67	98.16
	AlexNet	97.65	98.65	98.34	98.49
	Modified UNet	95.23	95.45	95.56	95.50
K = 4	VGG16	95.89	96.02	96.34	96.17
	VGG19	94.99	95	95	95
	AlexNet	96.08	95.04	95.22	95.12
	Modified UNet	98.2	98.34	98.34	98.06
K = 5	VGG16	98.88	98.45	98.56	98.50
	VGG19	97.45	98.56	97.56	98.05
	AlexNet	96.66	95.80	95.23	95.51
	Modified UNet	98.67	99.02	98.07	98.54

**Table 4:** Ratings of training variables used for segmentation and classification.

Constraints	Segmentation	Classification
Learning rate	0.002	0.002
Epochs patience	10	8
Batch size	16	16
Epochs	50	15
Loss function	NLL/BCE/MSE	BCE
Stopping criteria	8	5

**Table 5:** Performance analysis of classifiers without data augmentation for NLL loss function.

Case	Classifier without Data Augmentation	Accuracy (%)	Precision (%)	Recall (%)	F1-Score (%)
Case 1	VGG16	94.08	94.62	95.24	94.68
	VGG19	95.79	96.01	95.33	96.37
	AlexNet	92.36	92.64	93.46	94.29
	Modified UNet	97.11	98.25	97.06	98.32
Case 2	VGG16	93.86	94.11	94.82	93.95
	VGG19	95.47	95.45	96.08	95.91
	AlexNet	91.28	91.34	90.26	91.56
	Modified UNet	96.24	97.08	96.37	97.11
Case 3	VGG16	92.09	92.86	93.01	93.27
	VGG19	93.15	93.22	94.63	93.98
	AlexNet	90.68	91.26	90.08	91.72
	Modified UNet	94.56	96.31	95.94	96.94

**Table 6:** Performance analysis of classifiers with AS-HPOARA for NLL loss function.

Case	Classifier with AS-HPOARA	Accuracy (%)	Precision (%)	Recall (%)	F1-Score (%)
Case 1	VGG16	96.93	95.64	96.93	96.44
	VGG19	98.82	97.22	97.06	96.79
	AlexNet	95.08	95.93	94.28	94.77
	Modified UNet	99.08	98.74	99.21	99.03
Case 2	VGG16	96.05	95.08	96.7	96.17
	VGG19	97.84	97.08	97.46	96.63
	AlexNet	94.47	94.84	95.05	95.08
	Modified UNet	98.64	98.5	98.74	98.93
Case 3	VGG16	95.05	95.28	96.46	96.1
	VGG19	96.68	96.05	96.43	95.58
	AlexNet	93.35	94.58	94.58	93.09
	Modified UNet	98.2	98.34	98.34	98.06

**Table 7:** Comparative analysis of AS-HPOARA for Case 1.

Performance Metrics	Dataset	DCGAN-GoogleNet [21]	AS-HPOARA
Accuracy (%)	Tomato Plant Village dataset	94.33	99.08

**Table 8:** Comparative analysis of AS-HPOARA for Case 3.

Performance Metrics	Dataset	CGAN-DenseNet121 [22]	AS-HPOARA
Accuracy (%)	Tomato Plant Village dataset	97.11	98.7
Precision (%)		97	98.52
Recall (%)		97	98.58
F1-score (%)		97	98.27

**Table 9:** Comparative analysis of AS-HPOARA with existing RAHC_GAN.

Performance Metrics	Dataset	RAHC_GAN [27]	AS-HPOARA
Accuracy (%)	Tomato Plant Village dataset	98.1	99.7

## Data Availability

The datasets used during the current study are available from the corresponding author on reasonable request.
